# Spectrofluorimetric determination of butylated hydroxytoluene and butylated hydroxyanisole in their combined formulation: application to butylated hydroxyanisole residual analysis in milk and butter

**DOI:** 10.1038/s41598-024-54483-1

**Published:** 2024-02-24

**Authors:** Sara Abdel Basset Galal, Eman Saad Elzanfaly, Emad Mohamed Hussien, Enas Abdel Hakim Amer, Hala Elsayed Zaazaa

**Affiliations:** 1 Egyptian Drug Authority, 51 Wezaret El-Zeraa Street, Agouza, Giza, 12618 Egypt; 2 Analytical Chemistry Department, Faculty of Pharmacy, Cairo University, Kasr El-Aini Street, Cairo, 11562 Egypt

**Keywords:** Spectrofluorimetry, BHT, BHA, Dosage form, Milk, Butter, Analytical chemistry, Risk factors

## Abstract

Butylated hydroxytoluene (BHT) and butylated hydroxyanisole (BHA) are two antioxidants that have been extensively used in many applications. Both are well known for their debatable health risks due to their multiple intake sources. Therefore, conservative limits are set for them in different regulations adapted to the matrices in which they exist. Here we present a simple spectrofluorimetric method for the determination of BHT and BHA based on their native fluorescence and synchronous scanning mode. The type of solvent and the interval between emission and excitation wavelengths were carefully optimized. Under the optimized conditions, good linearities were obtained between the emission intensity and the corresponding concentrations of BHT and BHA over the range of 3–18 µg/mL and 0.1–7 µg/mL, respectively with a good correlation coefficient (r > 0.99). The limits of detection were 0.9 and 0.02 µg/mL, and the quantification limits were 3 and 0.05 µg/mL for BHT and BHA, respectively. The suggested procedure was validated according to ICH guidelines Q2 (R1). Furthermore, the method’s greenness was assessed by three different methods, and it proved to be eco-reasonable. The method was successfully applied to the determination of BHT and BHA in pharmaceutical formulations. We also applied the suggested method for monitoring the residual BHA in conventional, powdered milk and butter, with good recovery in spiked samples.

## Introduction

Butylated hydroxytoluene (BHT) and butylated hydroxyanisole (BHA) are two synthetic phenolic antioxidants that are extensively used in many applications, including food additives, cosmetics, personal care products, pharmaceuticals, plastics/rubbers, and other petroleum products. They act to disturb the oxidative chain reactions by scavenging free radical species, maintaining the properties and integrity of products that are susceptible to lipid oxidation when exposed to air, light, or any other trigger^1^.

BHT and BHA combinations show synergistic antioxidant effect^2,3^, so they are added either individually or in combination with each other or other antioxidants such as gallates or tertiary butylhydroquinone (TBHQ) to beverage whiteners, breakfast cereals, butter oil, anhydrous milk fat, ghee, chewing gum, cocoa and chocolate products, dried vegetables, seaweeds, nuts, seeds, fat spreads, dairy fat spreads and blended spreads, food supplements, frozen fish, fish fillets, and fish products, including mollusks, crustaceans, and echinoderms. In pharmaceutical preparations and cosmetic formulations, they act as preservatives and antioxidants for those preparations and formulations containing fats and oils. Both are added to animal feed at a limit of up to 150 mg/kg complete feed^4,5^, and this can be a source for their residual presence in animals producing meat or milk.

Due to their ubiquitous uses, concerns about their health effects have been raised in hundreds of publications, health, and food authorities’ reports. Their cumulative effect results from multiple sources of exposure and ingestion. Sufficient evidence of carcinogenicity from studies in experimental animals supported BHA to be reasonably anticipated as a human carcinogen by the national toxicology program in the USA, as mentioned in the report of carcinogens^6^. Chronic toxicity studies have been associated with the induction of benign and malignant tumors of the forestomach in rats and hamsters by administration through diet. It was found toxic to the reproductive system and embryo in rats not to pigs, rabbits, or rhesus monkeys^7^. Also, in October 1985, IARC, (International Agency for Research on Cancer) reviewed BHA and based on sufficient evidence of carcinogenicity in experimental animals, BHA was classified in Group 2B (Possible Human Carcinogen), but no data were available to support evidence of carcinogenicity in human beings^8^. BHA is classified and generally recognized as safe by the US Food and Drug Administration if the total antioxidant content (BHA alone or in combination with BHT) doesn’t exceed 0.02% w/w of the total fat or oil content of the food. In other specific products, a maximum level of 0.001–0.02% is allowed^7,9^.

Due to their susceptible controversial health hazards, they are strictly regulated by well-defined limits and specifications in many countries for their usage in different food products and pharmaceutical formulations. The literature survey revealed many articles for their quantitative determination in pharmaceuticals and food matrices. High performance liquid chromatography represents the key analytical tool for analysis due to its high sensitivity, selectivity, and affordability in many labs. BHT and BHA were either determined singly or most commonly simultaneously in different matrices (edible oils, fish, milk, cosmetics, biological fluids, butter, margarine and snacks) by High performance liquid chromatography coupled with UV detector^10–26^, fluorescence detector^26–29^, LC or GC coupled with mass spectrometry^26,30–36^, GC-FID^37–42^, GC-ECD^43^. BHT and BHA were also determined simultaneously by spectrophotometry in pharmaceutical preparations and chewing gums^44^. Spectrofluorimetric determination of Butylated hydroxyanisole and propyl gallate in foodstuffs was reported by derivatization with 4-chloro-7-nitrobenzofurazan (NBD-Cl)^45^. BHA was detected in foodstuffs by measuring the emission intensity at 323 nm upon excitation with a wavelength of 293 nm^46^. Spectrofluorimetric determination of BHA in foodstuffs was also presented based on the derivatization of a phenolic hydroxy group by dansylation^47^. The determination of BHA in food products and packaging material by spectrofluorimetry was also reported^48^. BHT was determined in food by fluorimetry based on its oxidation with KMnO_4_ solution to give a highly fluorescent compound^49^.

Based on the literature survey, no spectrofluorimetric method was presented for the simultaneous determination of BHT and BHA and due to its simplicity and higher sensitivity, the aim of the present work was to develop and validate a spectrofluorimetric method that can determine BHT and BHA in pharmaceutical formulations based on their native fluorescence. Binary mixtures of BHT and BHA could be resolved utilizing zero crossing synchronous spectrofluorimetry. The method was applied to simple and rapid determination of BHT and BHA in pharmaceutical formulation, and it could be successfully applied to monitoring the presence of BHA in powdered and liquid milk samples with good recovery of the spiked samples. It was also applied to the determination of BHA in butter by the standard addition method.

## Materials and methods

### Chemicals and reagents

All chemicals were of analytical grade, and solvents were of HPLC grade.

Butylated hydroxytoluene and butylated hydroxyanisole were kindly supplied from Al Andalous Medical Company with potency 99.9% and 99.8%, respectively.

Methanol, acetonitrile (Supelco Merck, Germany), sulfuric acid (Merck, Germany), ammonium sulphate (LOBA chemie, India), n-hexane (Carlo erba, France) water for injection (OTSUKA, Egypt) were used.

Zarotex gel (Al Andalous Medical Company) with a nominal concentration of BHT and BHA 0.5 mg/g was purchased from the local market.

### Instrumentation

Shimadzu spectrofluorophotometer RF-6000 (Kyoto,Japan) equipped with 150 W Xenon lamp and 1.0 cm quartz cell was used for spectrofluorimetric measurements. The spectral bandwidth for both monochromators was set at 5 nm with auto adjustment of the sensitivity of detector. The spectrofluorophotometer is connected to Lenovo computer loaded with LabSolutions RF software, Thermo Scientific™ Megafuge (UK). Sartorius Secura 324-1S analytical balance (Germany), vortex V2H (Boeco, Germany) and ultrasonic water bath (R. Espinar S.L, Spain) were used.

### Preparation of standard solutions of BHT and BHA

(i) *For calibration curves construction* standard stock solution of BHA and working standard solution of BHT were prepared at a final concentration of 250 µg/mL in 0.75% sulfuric acid in acetonitrile. BHA working standard solution of final concentration 100 µg/mL was prepared by appropriate dilution of its standard stock solution in the same solvent.
(ii) *For spiking milk and butter samples* working standard solution of BHA was prepared by an appropriate dilution of BHA standard stock solution in methanol to a final concentration of 20 µg/mL.

### Construction of calibration curves

Into two sets of 10 mL volumetric flasks, aliquot volumes of the working standard solutions for calibration curve equivalent to 30–180 µg of BHT and 1–70 µg of BHA were transferred, then completed to the mark with 0.75% sulfuric acid in acetonitrile and mixed well. The fluorescence emission was acquired between 222 and 400 nm with a data interval of 1 nm and a scan speed of 6000 nm/min with auto adjustment of the detector sensitivity against 0.75% sulfuric acid in acetonitrile. BHT and BHA were determined by measuring emission at 293 nm and 317 nm, respectively.

### Construction of calibration curves in milk samples

Two millilitres of liquid milk or one gram of powdered milk samples in which BHA gave no detectable spectrum were quantitatively transferred or weighed into 50 mL falcon tubes. Powdered milk samples were dissolved in 1.5 mL water for injection by vortexing for 30 s, then fortified with different aliquots of BHA working standard solution and left for 15 min to allow BHA absorption into the matrix. Milk proteins were then precipitated by adding 1.5 g ammonium sulphate; falcon tubes were vortexed for 30 s, left in the refrigerator for 30 min till complete precipitation, and then extracted with 6 mL acetonitrile by vortexing for 2 min. Samples were centrifuged at 13,000 RPM and 4 °C for 15 min. 0.75 mL of the clear supernatant was diluted to 5 mL with 0.75% sulfuric acid in acetonitrile. Samples were measured as per the aforementioned procedures under 2.4 against blank milk samples without spiking.

### Assay of BHT and BHA in their pharmaceutical dosage forms

An accurately weighed one gram of Zarotex gel was weighed into 50 mL stoppered conical flask, dissolved in 10 mL 0.75% sulfuric acid in acetonitrile in an ultrasonic water bath for 10 min, transferred quantitatively into 25 mL volumetric flask, with rinsing the flask thoroughly with solvent, completed to the volume with 0.75% sulfuric acid in acetonitrile and mixed well. Into two 5 mL volumetric flasks, 0.2 mL and 2.5 mL were separately diluted to 5 mL with 0.75% sulfuric acid in acetonitrile, and the above procedures were followed as mentioned under “2.4. Construction of calibration graphs”.

### Analysis of butter sample by liquid -liquid extraction and standard addition technique

One gram of butter was weighed separately into five 50 mL falcon tubes, spiked with different concentrations of BHA from BHA working standard solution (2.5, 5, 7.5, 10 µg/g). Samples were vortexed for 30 s, dissolved in 1 mL hexane by vortexing for 1 min, and extracted with 4 mL acetonitrile by vortexing for 5 min. All samples were centrifuged at 13,000 RPM at 4 °C for 5 min.

In a series of five 5 mL volumetric flasks, 0.3 mL of the clear supernatant was diluted to 5 mL with 0.75% sulfuric acid in acetonitrile. Samples were measured as per the aforementioned procedures under 2.4.

## Results and discussion

Both BHT and BHA are native fluorescent molecules, with a higher emission intensity for BHA compared to BHT at the same concentration. This triggered us to try to develop a sensitive, eco-friendly, and simple spectrofluorimetric method for their determination utilizing synchronous spectrofluorimetric scanning mode for resolving both analytes’ spectra, thus enhancing selectivity while maintaining good sensitivity.

### Method optimization

#### Selection of the optimum wavelength interval (∆λ)

**Figure 1:**
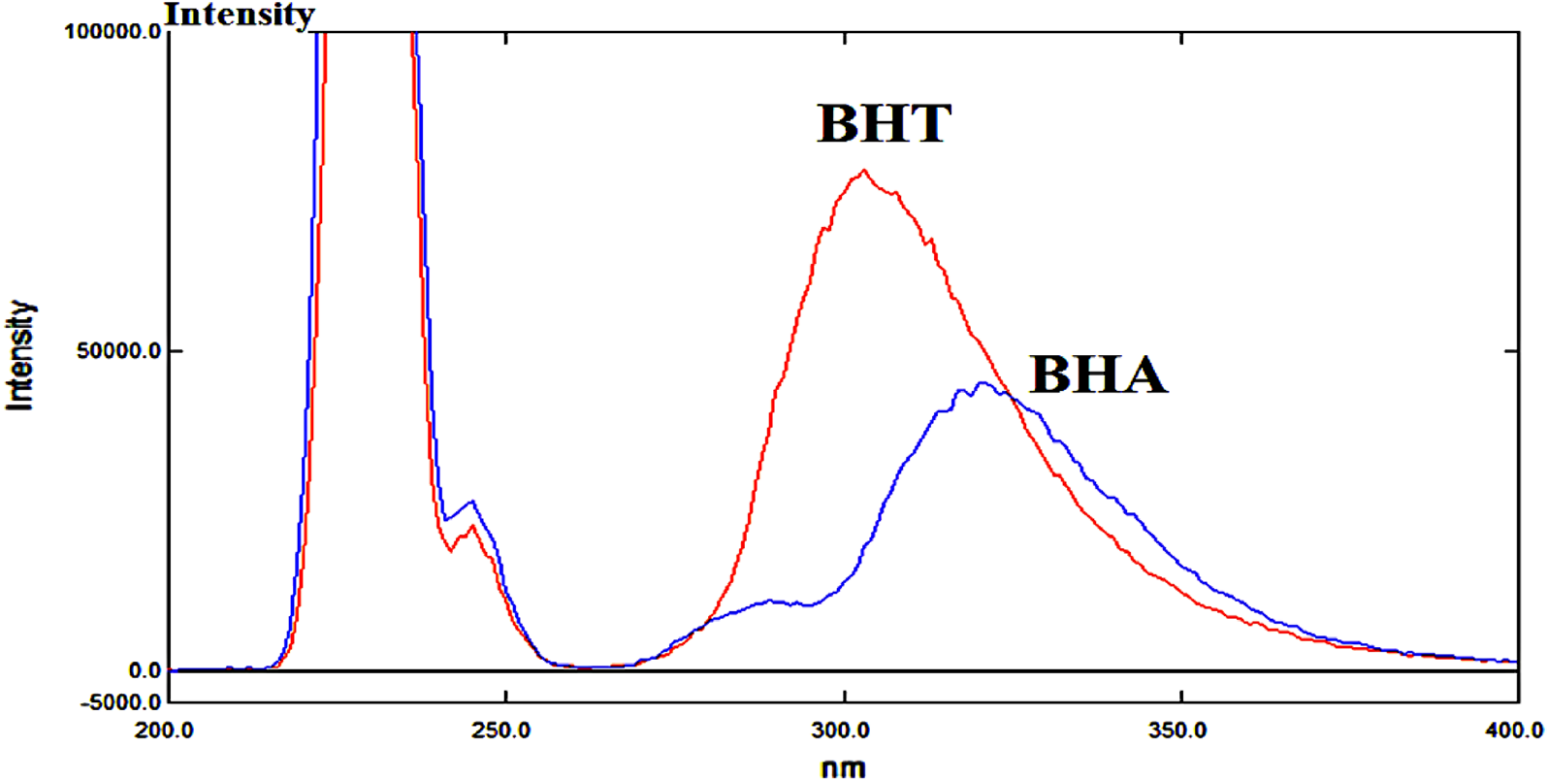
Overlapping spectra of BHT (12 µg/mL) and BHA (0.3 µg/mL) in zero order fluorescence spectra (excitation wavelength 220 nm, excitation and emission band widths 5 nm, Scan speed 6000 nm/min).

**Figure 2:**
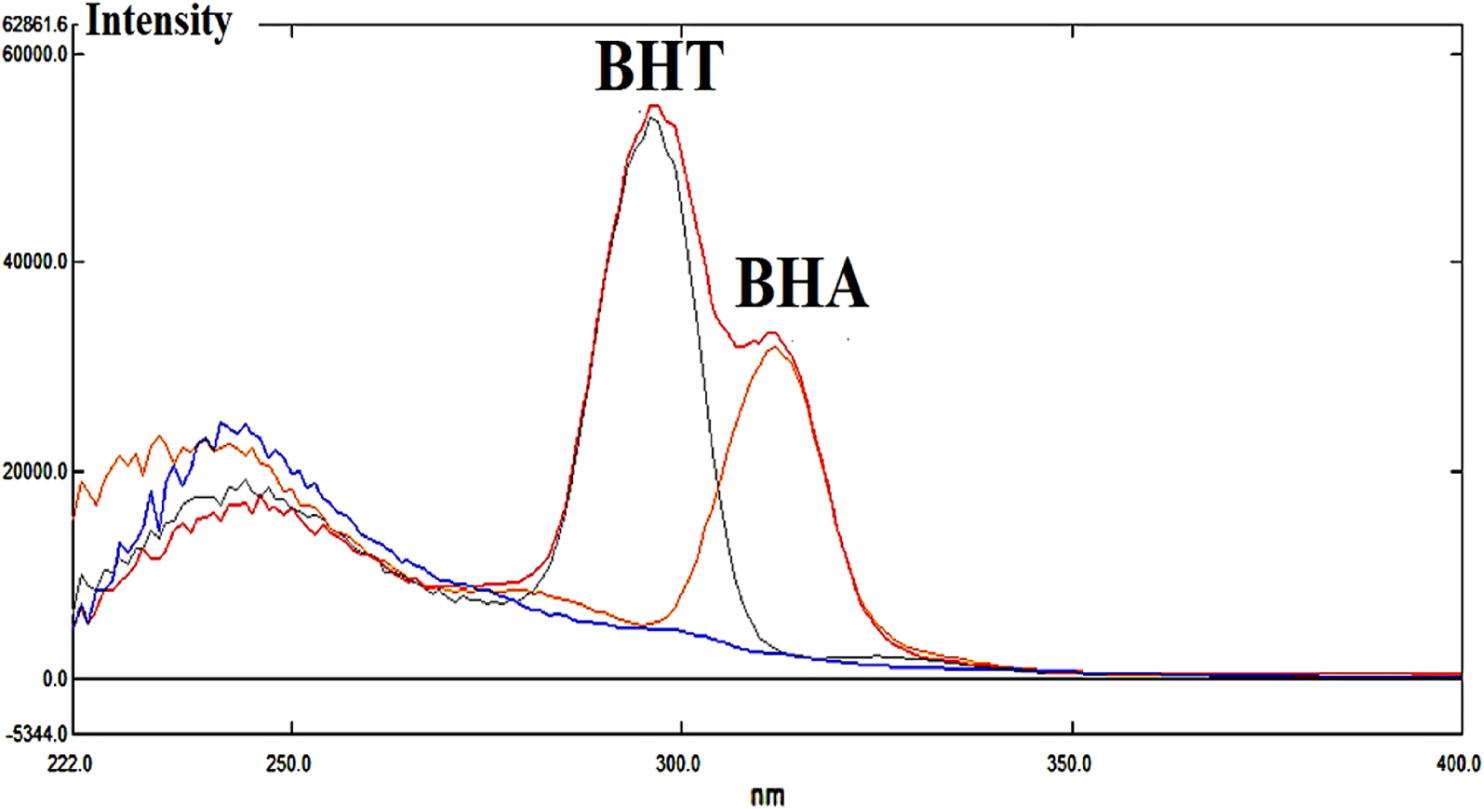
Schematic diagrams of synchronous fluorescence spectra in 0.75% sulfuric acid in acetonitrile (BHT (10 µg/mL)–BHA (0.35 µg/mL)–Mixture–Blank spectra) under optimized conditions (∆λ = 22, Excitation, and emission band widths 5 nm, Scan speed 6000 nm/min).

Both BHT and BHA show considerable overlap between their spectra in zero order fluorescence upon excitation with different wavelengths (Fig. 1). Synchronized scan mode allows scanning samples by both monochromators (excitation monochromator and emission monochromator) simultaneously with an offset of fixed wavelength intervals. Sharp fluorescence peaks with good resolution between the emission spectra of substances in mixtures can be obtained by optimizing wavelength offset or interval (∆λ). So, we considered several wavelengths’ intervals. It was found that ∆λ = 22 gave sensitive, well resolved spectra for both BHT and BHA. The emission wavelengths were 293 nm and 317 nm for BHT and BHA, respectively with zero crossing for each substance at the measurement wavelength of the other. Excitation and emission band widths were set to 5 nm as a good compromise between sensitivity and background noise, with auto adjustment of detector sensitivity and scan speed 6000 nm/min (Fig. 2).

#### Optimization of the effect of diluting solvent

Solvents of different polarities have dramatic effects on both the intensity and resolution of the emission spectra of different substances. Since BHT exhibits remarkable hydrophobicity and lower aqueous solubility, and BHA is also water insoluble, several organic solvents of different polarities were evaluated for their effect on BHT and BHA emission spectra and intensity. Dilution with methanol–ethanol–isopropanol–acetonitrile–butanol–heptane showed that methanol–ethanol–isopropanol had comparable effects regarding emission intensity and resolution between both BHT and BHA spectra. Butanol showed a remarkable reduction effect on the emission intensities of both BHT and BHA. Heptane reduced the emission intensity of BHA while BHT emission in heptane was comparable to its value in methanol–ethanol– and isopropanol. Acetonitrile gave the highest sensitivity and the best resolution between BHT and BHA spectra. So, it was chosen for further spectrofluorimetric measurements.

**Figure 3:**
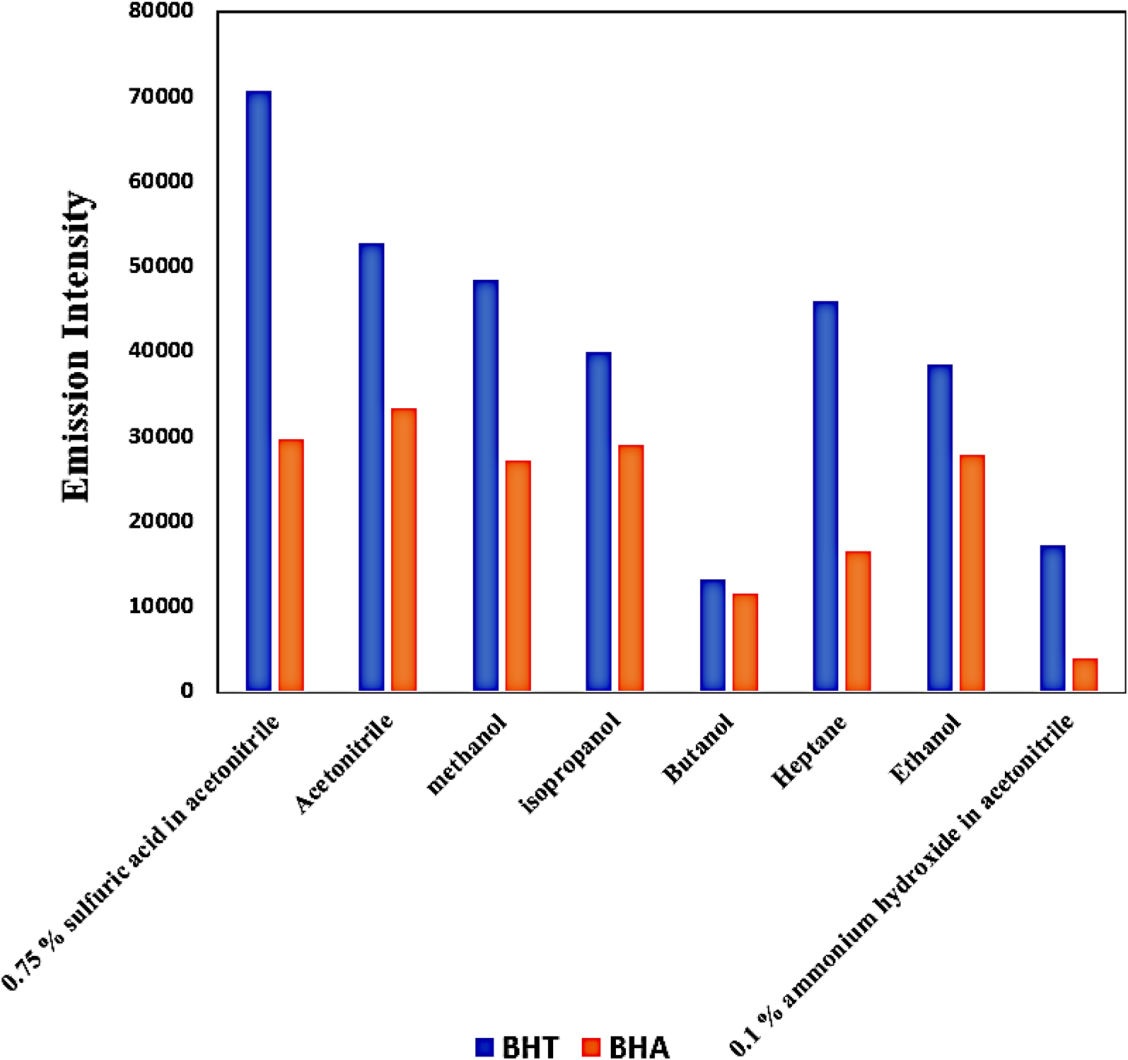
Effect of different diluting solvents on BHT and BHA emission intensity (BHT concentration 12.5 µg/mL–BHA concentation 0.25 µg/mL) under experimental conditions (∆λ = 22, Excitation, and emission band widths 5 nm) at room temperature in 3.5 mL standard cuvette volume.

Affecting the medium acidity by adding sulfuric acid to acetonitrile further enhanced emission intensity remarkably for BHT with minor enhancement in spectra resolution between both BHT and BHA. Several percentages of sulfuric acid in acetonitrile were tested where increasing percentages of sulfuric acid in acetonitrile enhanced fluorescence intensity for BHT with a minor reduction in emission intensity of BHA till 0.75% of sulfuric acid in acetonitrile after which increasing percentage of sulfuric acid in acetonitrile reduced emission intensity for both BHT and BHA. So, 0.75% of sulfuric acid in acetonitrile gave the optimum intensity with the lowest background noise and was the solvent of choice for further measurements. On the other hand, the addition of ammonium hydroxide to acetonitrile remarkably reduced the emission intensity of both BHT and BHA probably due to BHT and BHA instability at pH ≥ 9^50^. Acetonitrile also has an added advantage over other solvents such as ethanol and methanol in that it exhibits minimal solubility for fats, making it an excellent choice for liquid -liquid extraction of butter samples, also minimizing any interference from fats in milk samples’ extraction. The solubility effect of different solvents on the emission intensity of both BHT and BHA is illustrated in Fig. 3.

#### Optimizing milk proteins precipitation

We tested several approaches to remove interference due to milk proteins, precipitation by simply lowering milk pH by adding acid to reach isoelectric points of milk proteins and precipitation by ferrous sulphate and chitosan, but all approaches failed to give clear background upon measurement. Salting out of milk proteins with several salts was tested using Na citrate, Na sulphate, or sodium chloride. The lowest background noise was obtained by salting out with ammonium sulphate due to its high-water solubility giving the maximum salting out effect on milk proteins among the tested salts. It also decreased acetonitrile solubility in water to enhance the separation of the aqueous and organic phases from the extract and cleaned the acetonitrile phase from matrix components by salting out effect.

#### Optimizing the extraction process from milk and butter

We tested different time intervals for vortex assisted extraction of BHA from milk and butter. The efficiency of the extraction process was evaluated every time by comparing the detector response (emission) of amount of BHA pre-spiked and extracted from blank milk samples (pre-spiked samples) with non-extracted samples (post extraction spiked samples representing 100% recovery).

For butter sample, due to the already existence of BHA in butter and the absence of an appropriate blank matrix, we used the standard addition method. We confirmed the recovery obtained by comparing it with that obtained by applying the method reported by Saad et al.^16^.

### Validation of the proposed method

Validation of the method was carried out according to ICH guidelines Q2(R2), Guidance for Industry and Q2B of analytical procedure^51,52^.

#### Linearity

The linearity of the proposed method was evaluated by analysing a series of different concentrations of BHT and BHA in the chosen solvent. Linearity for BHA analysis in milk samples was investigated by matrix matched calibration curves (MMCC) in powdered and liquid milk where minimum seven milk calibration samples were prepared by fortification from BHA working standard solution at different levels, extracted, and analyzed.

The linearity of the developed method was estimated, and the linear regression data for the calibration curves showed good linearity (r ≥ 0.99) over the concentration range of 3–18 µg/mL and 0.1–7 µg/mL for BHT and BHA, respectively in the chosen solvent. For MMCC of BHA in powdered and liquid milk, both curves were linear with a correlation coefficient > 0.99 and a slope (with their confidence intervals (p = 95%) that did not include the 0 value) over concentration range of 2–30 mg/kg and 0.25–10 mg/L in case of powdered and liquid milk, respectively (calibration curves are provided in Supplementary Material).

#### Limits of detection and quantitation

The limit of detection (LOD) was established by determining the minimum level of the analyte that can readily be detected, while the limit of quantification (LOQ) was established as the level of the analyte below which the calibration graph is non‐linear.

Specific calibration curves were studied using samples containing BHT and BHA in the range of DL and QL. The standard deviation of the y-intercepts of regression lines was used as the standard deviation^51^.

The detection limit (DL) was expressed as:

$${\text{DL}} = { 3}.{3 }\sigma /{\text{S,}}$$

where σ is the standard deviation of the response, S is the slope of the calibration curve.

The quantification limit (QL) was expressed as:

$${\text{QL}} = { 1}0 \, \sigma /{\text{S}},$$

where σ is the standard deviation of the response, S is the slope of the calibration curve.

The limits of detection were 0.9 and 0.016 μg/mL, and quantification limits were 3 and 0.05 μg/mL for BHT and BHA respectively.

For BHA limit of quantification in milk matrix, it was taken experimentally as the lowest non-zero concentration in MMCC for which the standard deviation of 6 samples prepared by spiking blank samples does not exceed 20%. LOD was estimated as 3 times of the standard deviation.

The limits of detection were 0.7 mg/kg, and 0.1 mg/L, and the quantification limits were 2 mg/kg and 0.25 mg/L for BHA in powdered and liquid milk, respectively.

The proposed method showed good sensitivity with low limits of detection and quantitation for both BHT and BHA but with better sensitivity for BHA as it exhibits higher native fluorescence than BHT (Table 1).

**Table 1:** Analytical parameters for spectrofluorometric assessment of BHT and BHA.

Parameter	BHT (in acidified acetonitrile)	BHA (in acidified acetonitrile)	BHA (in powdered milk)	BHA (in liquid milk)
Wavelength (nm)	293	317	317	317
Linear range	3–18 µg/mL	0.1–7 µg/mL	2–30 mg/kg	0.25–10 mg/L
Determination coefficient	0.9995	0.9991	0.9961	0.9986
Intercept	10,979	6162.8	− 283.19	1538
Slope	4585.1	87,302	2028.2	24,545
LOD (mg/mL)	0.9 µg/mL	0.02 µg/mL	0.7 mg/kg	0.1 mg/L
LOQ (mg/mL)	3 µg/mL	0.05 µg/mL	2 mg/kg	0.25 mg/L
Repeatability (% RSD)	1.01^a^	1.92^a^	7.6^c^	6.24^c^
Intermediate precision (% RSD)	1.5^b^	1.95^b^	7.06^d^	6.62^d^
Concentration of pre-existing BHA in butter oil (mg/kg ± mg/kg)	–	8.64 mg/kg ± 0.4 mg/kg^e^	–	–

^a^Evaluation of six determinations at 100% concentration level in first day.

^b^Pooled RSD value for evaluation of six determinations at 100% concentration level in two different days.

^c^Evaluation of three determinations of fortified samples at three levels in first day.

^d^Pooled RSD value for evaluation of six determinations of fortified samples at three levels in two different days.

^e^Average of three determinations.

#### Accuracy

The accuracy of the method was studied by recovery experiments at three different concentrations covering the linearity range. The overall results are expressed as the percentage recovery of the added amount of analyte. Results were shown in (Table 2) indicating good accuracy of the proposed method. Accuracy was also confirmed by standard addition on dosage form (Tables 3, 4).

**Table 2:** Evaluation of the accuracy of the proposed spectrofluorimetric method in 0.75% sulfuric acid in acetonitrile.

BHT		BHA	
Nominal concentration taken (µg/mL)	% Recovery ± % RSD*	Nominal concentration taken (µg/mL)	% Recovery ± % RSD*
3	101.78% ± 1.80	0.25	100.4% ± 0.92
8	100.58% ± 0.39	3	99.92% ± 0.74
16	99.56% ± 0.71	6	99.18% ± 0.97

*Average of three determinations.

**Table 3:** Evaluation of the accuracy of the proposed spectrofluorimetric method for the determination of BHT by standard addition on Zarotex® gel.

Claimed conc of BHT taken from gel in (µg/mL)	%Found conc. ± RSD*	Std. conc. added (µg/mL)	Total recovered conc (µg/mL)	Recovered conc of standard added (µg/mL)*	% Recovery*
10	94.46% + 1.50	4	13.49	4.04	101.00
		5	14.38	4.93	98.60
		8	17.12	7.67	95.88

*Average of three determinations.

**Table 4:** Evaluation of the accuracy of the proposed spectrofluorimetric method for the determination of BHA by standard addition on Zarotex® gel.

Claimed conc of BHA taken from gel in (µg/mL)	%Found conc ± RSD*	Std. conc. added (µg/mL)	Total recovered conc (µg/mL)	Recovered conc of standard added (µg/mL)*	% Recovery*
0.8	93.00% ± 1.50	1	1.75	1.01	100.63
		2	2.77	2.02	101.24
		4	4.72	3.98	99.52

*Average of three determinations.

For accuracy in the milk matrix, it was calculated as the recovery of BHA from fortified samples and the relative error in percentage between the nominal concentration value and the concentration obtained using the calibration curve < 15% for three milk samples fortified at three levels (LOQ, medium, and high concentrations in the calibration range) prepared in triplicate (Table 5).

**Table 5:** Evaluation of the accuracy and intermediate precision of the proposed spectrofluorimetric method in fortified milk samples.

BHA in powdered milk			BHA in liquid milk		
Fortified concentration (mg/kg)	Mean concentration recovery (mg/kg) ± mg/kg*	% Relative error	Fortified concentration (mg/L)	Mean Concentration Recovery (mg/L) ± % mg/L*	% Relative error
2	2.06 ± 0.21	3.00	0.25	0.23 ± 0.01	− 8.00
17.50	16.36 ± 0.51	− 6.51	3	2.91 ± 0.09	− 3.00
30	28.76 ± 1.57	− 4.13	7	7.35 ± 0.23	5.00

*Average of six determinations.

#### Precision

Samples of pharmaceutical formulation were analyzed over different days to obtain inter-day precision (intermediate precision, minimum of 6 determinations at 100% of the test concentration) and within the same day to obtain intra-day precision (repeatability) then the RSDs % values were calculated. The results of repeatability and intermediate precision experiments are shown in (Table 1). The developed methods were found to be precise, as the RSD% was < 2%.

The precision of the method in milk matrix was evaluated by calculating the relative standard deviation for recovery results of fortified samples in accuracy (repeatability) and on two different days (intermediate precision and intermediate accuracy) with acceptance criteria ˂ 15% (Table 5).

#### Selectivity

The selectivity of the proposed method was confirmed by applying the standard addition on the dosage form to which known amounts of BHT and BHA were added to give good recovery results. The selectivity results are summarized in (Tables 3, 4).

#### Analysis of BHT and BHA in Zarotex® gel

The suggested method was successfully applied for the determination of BHT and BHA in Zarotex® Gel. The results were in good agreement with the labelled amount. Application of the standard addition technique reveals no interference due to excipients in dosage form (Tables 3, 4).

#### Application for determination of BHA in milk samples and butter

The established limits for BHA in General Standard for Food Additives (GSFA) are 100 mg/kg for milk and 175 mg/kg in butter oil on the fat or oil basis singly, or in combination with butylated hydroxytoluene and propyl gallate^53^. Since the method showed good sensitivity, it could be successfully applied for monitoring the presence of BHA in real milk samples. The extraction procedures were applied as demonstrated under 2.5, and the corresponding concentrations were computed from the corresponding MMCC. We considered three powdered milk samples from the local market and one conventional milk sample. BHA wasn’t detected in one powdered milk sample, while the other two samples contained 2.76–3.35 mg/kg BHA, and the tested conventional liquid milk sample was found to contain 0.33 mg/L.

Also, the efficiency of the extraction process was evaluated by comparing the emissions obtained from pre-spiked and post spiked samples with known amounts of BHA, as shown in Tables 6 and 7.

**Table 6:** Evaluation of extraction efficiency of BHA from spiked powdered milk samples.

Concentration (µg/g)	%Recovery (pre-extraction/post extraction × 100)*	% RSD
2	92.98	2.44
3	93.76	3.65
6	95.20	3.51

*Average three determinations.

**Table 7:** Evaluation of extraction efficiency of BHA from spiked conventional liquid milk samples.

Concentration (µg/mL)	%Recovery (pre-extraction/post extraction × 100)*	% RSD
0.50	93.57	3.67
1	96.12	2.21
3	92.89	1.76

*Average three determinations.

**Figure 4:**
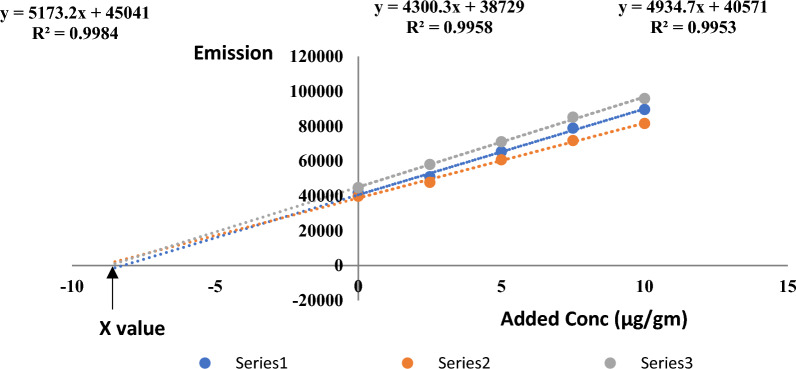
Quantification of BHA in butter oil sample by standard addition approach (X value represents the pre-existing BHA in butter sample).

We determined the amount of pre-existing BHA in butter oil by the standard addition method, where the recovered concentration was confirmed by comparing the recovery to that obtained by applying the method proposed by Saad et al.^16^ (Fig. 4).

Under the optimized conditions, the x value when y = 0 represents the amount of BHA actually present in the sample. Also, we constructed a calibration curve of the same added concentrations in solvent and its x intercept when y = 0 was subtracted from the x value of that obtained from spiked butter samples (almost trivial value). Results are shown in Table 1.

## Greenness assessment of the proposed method for the determination of BHT and BHA

In response to the mandatory concern to consider the greenness of any developed procedures, we assessed the greenness of the proposed method by three metrics, namely Analytical Eco-Scale Assessment (ESA)^54,55^, the Green Analytical Procedure Index (GAPI)^56^, and the last one: AGREE—Analytical GREEnness metric^57^.

Examination of method greenness by eco-scale metric showed that the method has an excellent greenness profile for analysis in dosage form and milk matrices, while it is eco-accepted for analysis of BHA in butter samples.

GAPI can also give a wider sight of the overall analytical procedure, starting from sample collection to waste treatment. From the pictograms of the three matrices, it can be seen that the red segments are due to the macroextration scale, the use of hexane being non-green solvent, the less eco-favorable acetonitrile, and the associated health and safety hazards also the absence of any treatment for the waste generated. The same information can be obtained in another way from AGREE twelve sections’ pictogram with additional data about the amount of sample used, analysis throughput, process automation, and operator safety. The red parts of the pictograms are due to the manual processing, hexane as a non-green solvent, but the method has the advantage of lower energy consumption inherent in spectrofluorimetry as a technique over the intense energy using LC-mass spectrometry instruments and higher sample throughput represented by a larger sample analyzed per unit time. Lower scores are noticed for BHA analysis in butter in all metrics due to the usage of hexane in butter solubilization. The results of greenness assessment are summarized in Tables 8 and 9 showing that the method is generally eco-accepted.

**Table 8:** Penalty points for greenness assessment of the proposed method by ECO scale tool.

Hazard	Penalty points (dosage form)	Penalty points (milk)	Penalty points (butter)
Acetonitrile	8	8	8
Sulphuric acid	2	2	2
Ammonium sulphate	–	0	–
Hexane	–	–	8
Instruments energy	0	0	0
Occupational hazard	0	0	0
Waste	8	8	8
Total penalty points	18	18	26
Analytical eco-scale total score*	82	82	74

*> 75 represents excellent green analysis, > 50 represents acceptable green analysis, < 50 represents inadequate green analysis.

**Table 9:** Pictograms of the greenness assessment of the developed method using GAPI and AGREE tools.

Representative pictogram	Dosage form	Milk	Butter
GAPI^a^			
AGREE^b^			

^a^The colour of the pictogram sections are green, yellow, and red indicating the low, medium and high environmental effect for each step of the procedures.

^b^The pictogram has total score of fraction of unity in the middle of the pictogram with values approaching 1 proving greener procedures and the number in each of the twelve sections of the pictogram referring to the criterion under investigation, each section’ width indicates their importance and the range of colour from deep green to deep red indicates the performance of the each criterion.

## Conclusion

A simple and economical spectrofluorimetric method was developed for the determination of BHT and BHA in their bulk powder and pharmaceutical formulation. The method was applied to monitor residual BHA in milk samples and butter based on the exceptional sensitivity and simplicity of spectrofluorimetry as an analytical technique over the standard, usually used chromatographic methods. The method proved to be accurate, selective, and sensitive making it suitable for routine quality control analysis and monitoring of residual BHA in milk and butter with satisfactory recovery with respect to the complexity of the studied matrices and the inherent variation in instrumental analysis.

### Supplementary Information

Supplementary Information.

## Data Availability

All data generated are included in the paper.
