# Supplementary figures and images for: Spectrofluorimetric determination of butylated hydroxytoluene and butylated hydroxyanisole in their combined formulation: application to butylated hydroxyanisole residual analysis in milk and butter
Source: Sci Rep. 2024 Feb 24;14:4498. doi: 10.1038/s41598-024-54483-1 (PMC10894300; doi:10.1038/s41598-024-54483-1)

**Emission**

### BHT Linearity in acetonitrile

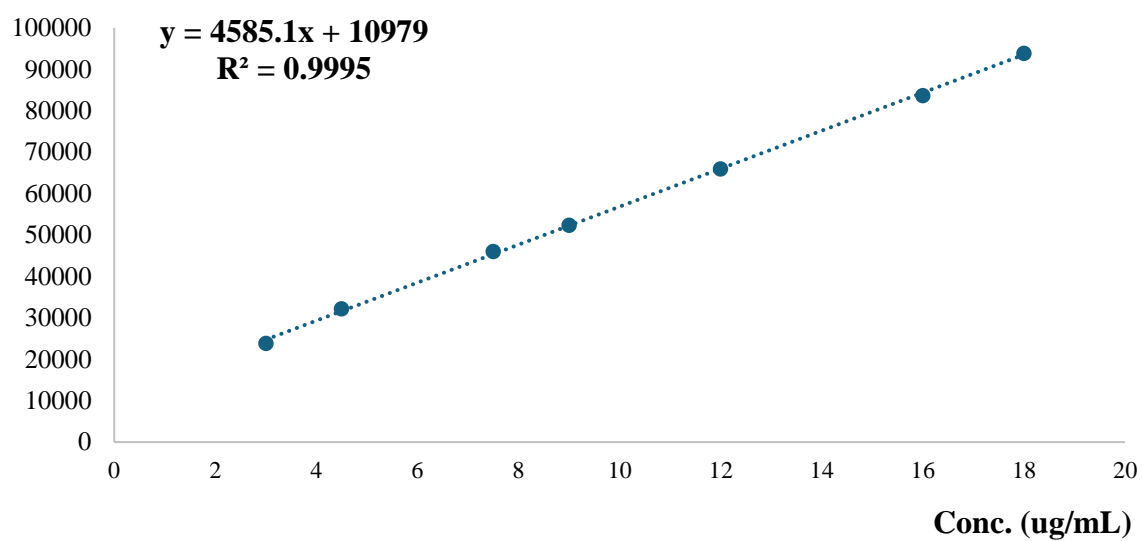

**Emission**

### BHA Linearity in acetonitrile

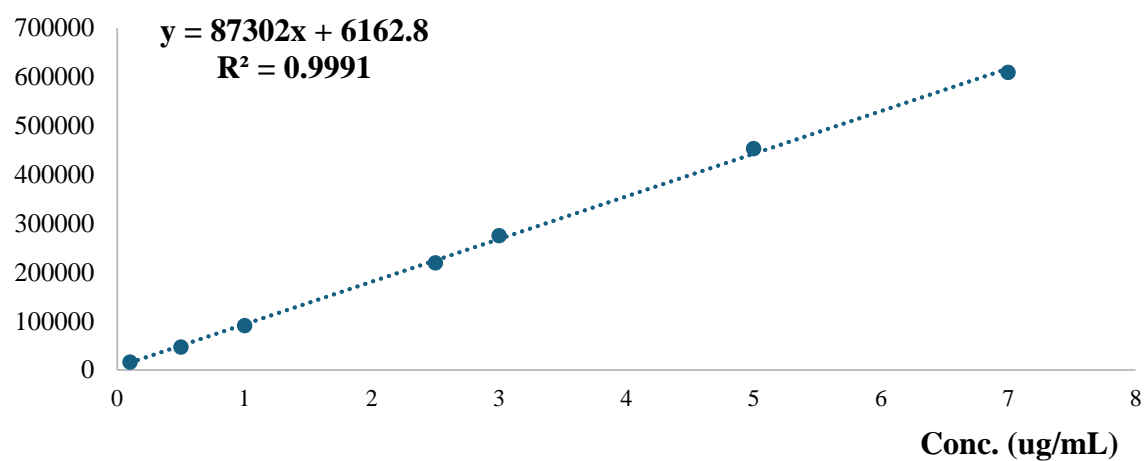

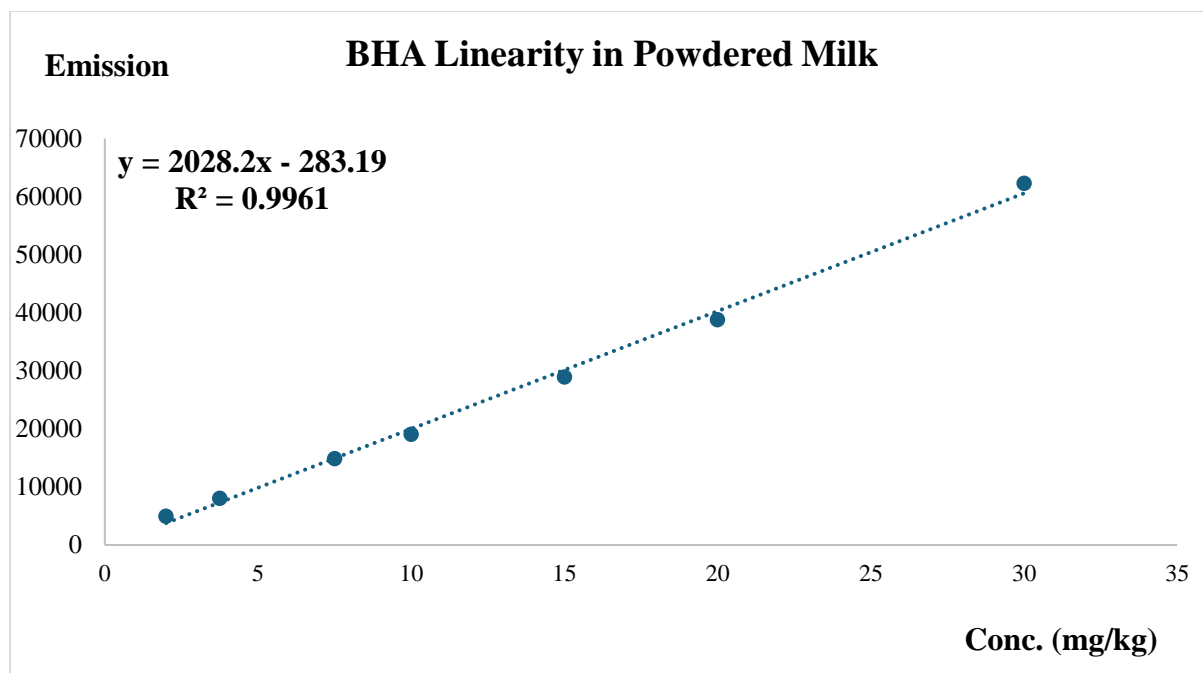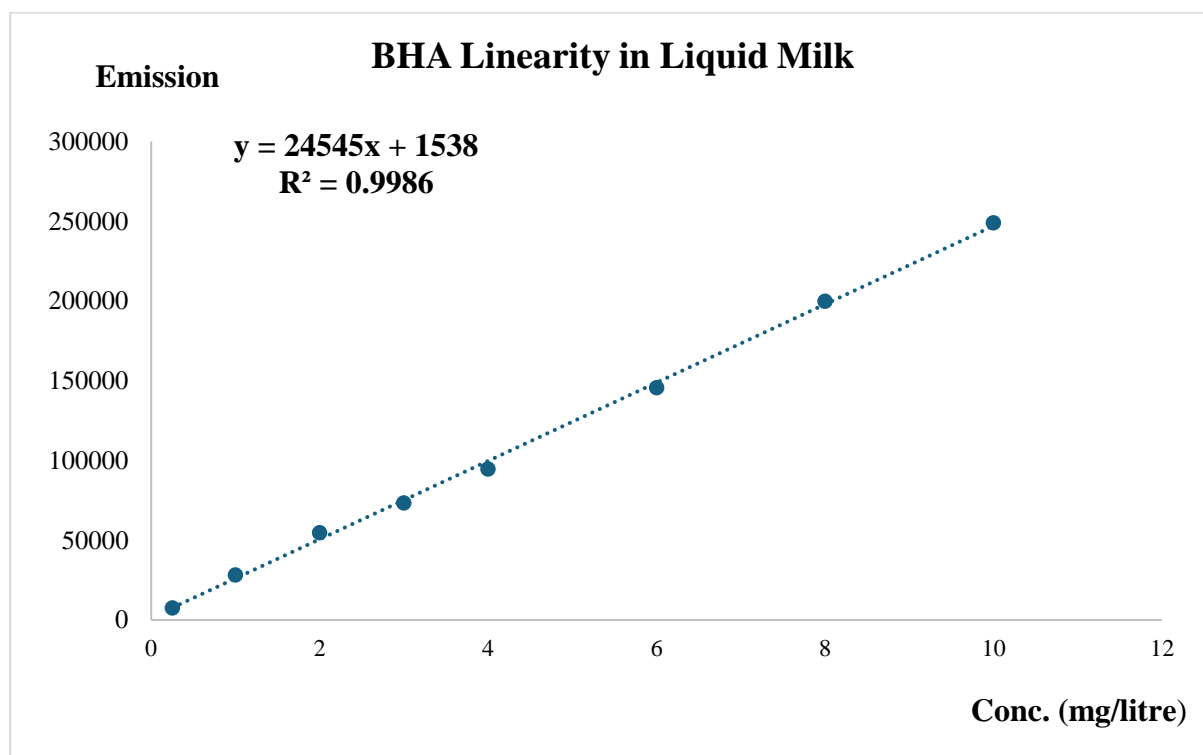

Supplement: Supplementary file 1 — Supplementary Information. [file 41598_2024_54483_MOESM1_ESM.pdf]
